# Middle and Later Stone Age chronology of Kisese II rockshelter (UNESCO World Heritage Kondoa Rock-Art Sites), Tanzania

**DOI:** 10.1371/journal.pone.0192029

**Published:** 2018-02-28

**Authors:** Christian A. Tryon, Jason E. Lewis, Kathryn L. Ranhorn, Amandus Kwekason, Bridget Alex, Myra F. Laird, Curtis W. Marean, Elizabeth Niespolo, Joelle Nivens, Audax Z. P. Mabulla

**Affiliations:** 1 Department of Anthropology, Harvard University, Peabody Museum of Archaeology and Ethnology, Cambridge, Massachusetts, United States of America; 2 Turkana Basin Institute and Department of Anthropology, Stony Brook University, Stony Brook, New York, United States of America; 3 National Museum of Tanzania, Shaaban Robert Street, Dar es Salaam, Tanzania; 4 Department of Human Evolutionary Biology, Harvard University, Peabody Museum of Archaeology and Ethnology, Cambridge, Massachusetts, United States of America; 5 Department of Organismal Biology and Anatomy, University of Chicago, Chicago, Illinois, United States of America; 6 Institute of Human Origins, School of Human Evolution and Social Change, Arizona State University, Tempe, Arizona, United States of America; 7 African Center for Coastal Palaeoscience, Nelson Mandela University, Port Elizabeth, South Africa; 8 Department of Earth and Planetary Science, University of California, Berkeley, California, United States of America; 9 Berkeley Geochronology Center, Berkeley, California, United States of America; 10 Center for the Study of Human Origins, Department of Anthropology, New York University, New York, New York, United States of America; Max Planck Institute for the Science of Human History, GERMANY

## Abstract

The archaeology of East Africa during the last ~65,000 years plays a central role in debates about the origins and dispersal of modern humans, *Homo sapiens*. Despite the historical importance of the region to these discussions, reliable chronologies for the nature, tempo, and timing of human behavioral changes seen among Middle Stone Age (MSA) and Later Stone Age (LSA) archaeological assemblages are sparse. The Kisese II rockshelter in the Kondoa region of Tanzania, originally excavated in 1956, preserves a ≥ 6-m-thick archaeological succession that spans the MSA/LSA transition, with lithic artifacts such as Levallois and bladelet cores and backed microliths, the recurrent use of red ochre, and >5,000 ostrich eggshell beads and bead fragments. Twenty-nine radiocarbon dates on ostrich eggshell carbonate make Kisese II one of the most robust chronological sequences for understanding archaeological change over the last ~47,000 years in East Africa. In particular, ostrich eggshell beads and backed microliths appear by 46–42 ka cal BP and occur throughout overlying Late Pleistocene and Holocene strata. Changes in lithic technology suggest an MSA/LSA transition that began 39–34.3 ka, with typical LSA technologies in place by the Last Glacial Maximum. The timing of these changes demonstrates the time-transgressive nature of behavioral innovations often linked to the origins of modern humans, even within a single region of Africa.

## Introduction

East Africa is central to understanding the biological and behavioral origins of modern humans, because of the presence of multiple early fossils attributed to *Homo sapiens* and early examples of Middle Stone Age (MSA) technology associated with them [1–3]. In part because of its geographic proximity to potential exit points along the Nile Valley and Horn of Africa, the region also features prominently in discussions of human population dispersals across and out of Africa by approximately 65,000 years ago (~65 ka) if not before [4–7]. However, our understanding of the social and environmental contexts of modern human populations in East Africa since ~65 ka remains poorly resolved due to a number of issues, prominent among them being the rarity of well-dated archaeological sequences that include terminal MSA and early Later Stone Age (LSA) assemblages (reviewed in [3, 8]). Broadly, the MSA/LSA transition consists of a series of technological and behavioral changes such as artifact miniaturization, expanded dietary breadth, and the increased use of symbolic artifacts and non-local materials that have been correlated with the origin of modern human cognitive abilities [9], increases in local population size, density [2, 10, 11] or inter-connectedness, potentially linked with environmental change [12, 13], and human dispersals [14].

**Fig 1:**
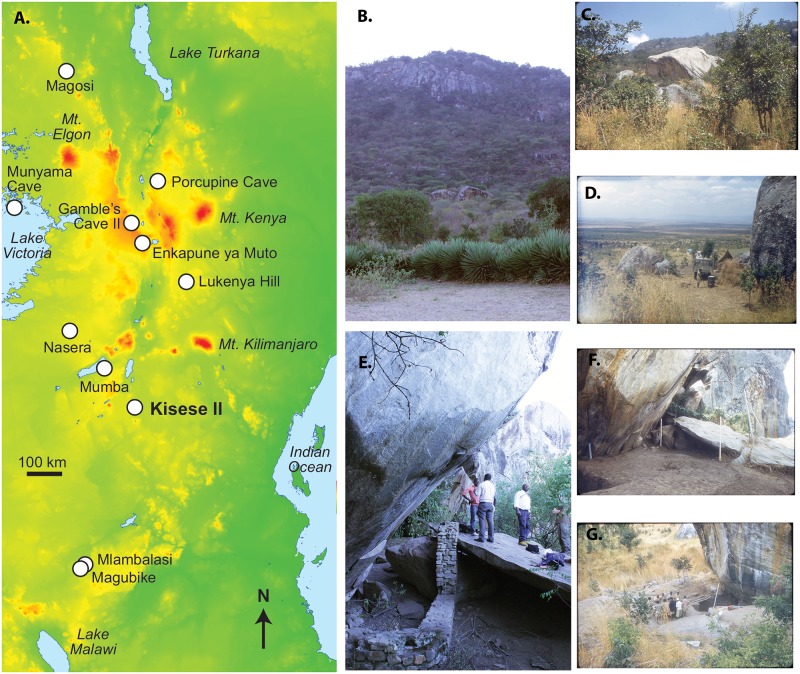
Kisese II in East African context. (A) Schematic map of East Africa and archaeological sites discussed in text, (B) boulders that form Kisese II below the Irangi Hills as seen in 2015, with arrow pointing to site, (C) view over the Masaai Steppe from Kisese II in 1956, (D) the 1956 Inskeep excavations in progress, (E) view of the shelter in 2015 with retaining wall, and (F) North excavation wall during the 1956 excavation; white labels record spit (level), with spit XX is the base of the excavation at the time the photograph was taken. Spit I (59 cm) and spit II (21 cm) are thicker than the remainder, which are each ~15-cm-thick. 1956 photographs by R. Inskeep (published with permission of J. Charlson, executor of the Inskeep estate), 2015 photographs by C. Tryon.

For East Africa in particular, no single archaeological sequence has been able to satisfactorily address whether the MSA-LSA transition is the summation of long-term, incremental changes in human behavior or a rapid behavioral revolution spurred by genetic or cognitive change as has been debated for nearly two decades [2, 9]. Available data have been used to suggest that the East African MSA-LSA transition was a complex, incremental process spanning 15 kyr or less beginning as early as 55 ka [3], but this hypothesis is based on comparisons across a series of poorly dated and irregularly described sequences. The small East African sample of sites with published and well-described MSA-LSA sequences (Fig 1) includes Magosi in Uganda, Enkapune ya Muto and GvJm22 (Lukenya Hill) in Kenya, and Mumba, Nasera, Mlambalasi and Magubike rockshelters, and perhaps Olduvai Gorge in Tanzania [15–19]. In addition to unconformities and incompletely understood sedimentary histories, the site sample has poor chronological resolution due to numerous infinite radiocarbon (^14^C) age estimates or other “dates” using unreliable materials and excavation methods that frequently mixed discrete strata. Mumba rockshelter has a robust chronology based on ^14^C and amino acid racemization dates on ostrich eggshell (OES) and optically stimulated luminescence ages on sediments, and its archaeological sequence is one of the most important in the region [2, 17, 20]. However, it is also one of the most difficult to interpret, as various parts of the cave have been differentially sampled and reported by four different teams operating at the site since the 1930s, with divergent interpretations based on temporal changes in lithic technology used to support hypotheses of either rapid *or* gradual shifts across the MSA/LSA transition [17, 18, 21, 22].

We present here new chronological and archaeological data from the vertically thick (~6 m) sedimentary succession at Kisese II rockshelter in north-central Tanzania, with 25 accelerator mass spectrometry (AMS) and four conventional ^14^C dates from the upper 3 m spanning 47–4 ka calibrated radiocarbon years before present (cal BP). The site is significant for its large sample (*n* >5,000) of early OES beads and evidence for ochre processing throughout much of its stratigraphic sequence. Both OES beads and ochre (particularly when used to color the surfaces of objects, walls, or bodies) are central to discussions about the origins of symbolic and aesthetic uses of material culture because of their role in generating, maintaining, and navigating personal and social identities, evidenced in ethnographic and historic records [13, 23–29]. Lithic artifacts associated with the OES beads and ochre at Kisese II include typical Middle Stone Age (MSA) forms (e.g., Levallois flakes and cores) as well as those common at Later Stone Age (LSA) sites (e.g., backed microliths and <2-cm-long bladelets). The stratigraphic patterning of different tool types and technologies, combined with our ^14^C chronology, suggests that Kisese II samples the MSA/LSA interface or transition and thus provides the foundation to assess the timing and tempo of the MSA/LSA transition in East Africa.

### Site context and excavation history

Kisese II (4°29'30.47"S, 35°48'43.31"E) is a painted rockshelter within the UNESCO World Heritage Kondoa Rock-Art Sites, a region of 2,336 km^2^ that contains the richest record of hunter-gatherer and agro-pastoralist rock art in East Africa [30–33]. The site consists of an east-facing overhang on one of two large (>100 m^3^) adjoining boulders ~200 m below the escarpment of the Irangi Hills (Fig 1), the eastern margin of the Gregory Rift Valley. These boulders and the shelter beneath them are a locally prominent and readily visible landscape feature (Fig 1). Mean annual precipitation in the area today is ~850 mm. *Brachystegia*-dominated drier miombo woodland occurs on the Irangi Hills, seasonally waterlogged *mbuga* soils are found at the base of the escarpment supporting wooded and edaphic grasslands, with drier *Acacia-Commiphora* bushland and thicket further to the east [34, 35]. Similar vegetation types occur in nearby Tarangire National Park, and support populations of waterbuck, wildebeest, hartebeest, lesser kudu, eland, buffalo, rhino, elephant, warthog, ostrich, and lion among others [36–38].

**Fig 2:**
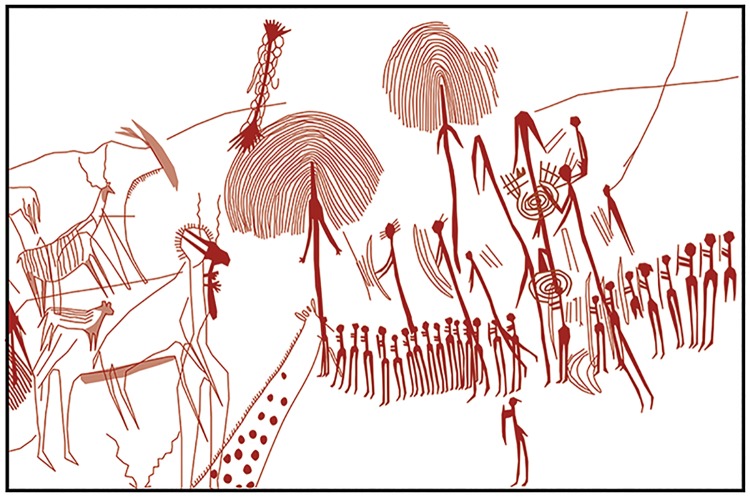
Rock art at Kisese II. Schematic representation of a portion of the painted rock face at Kisese II, redrawn from Leakey [31].

Archaeological investigations at the site began in 1935 when Louis and Mary Leakey visited it to study and document the rock paintings [31, 39–41], which include humans, animals (especially giraffe), and geometric figures executed in red and white (Fig 2). With the aim to date the rock art, the Leakeys excavated a ~5.3 m^2^ trial trench to a depth of 4 m in 1951 [41] but no details were published. At their invitation, Raymond Inskeep expanded the initial trial trench in 1956, excavating 21.3 m^2^ in generally silty sediments to a depth of ~6 m. Inskeep’s excavations successfully located *in situ* painted, exfoliated slabs of the shelter wall, recovered a large sample of well preserved and taxonomically diverse fossil fauna, thousands of ostrich eggshell beads and >5,900 lithic artifacts. Only a very brief summary of the excavation was published [42]. The full site report was delayed at least in part by Inskeep’s shift in academic focus to southern Africa beginning in 1957–1959 [43]. A note published in 1964 [44] on the age of Kisese II (on which Inskeep was not an author) failed to include the stratigraphic position of four conventional ^14^C measurements on burnt OES fragments. The stratigraphic uncertainty of the dated specimens effectively removed Kisese II from seminal discussions about the timing and tempo of African prehistory [45–48]. Although Inskeep returned to working on the Kisese II material in the 1980s, these efforts were abandoned with the theft of many of his field notes from his home in the 1990s.

Jim Simons examined portions of the faunal assemblage in the 1960s, as did Curtis Marean and Celeste Ehrhardt in the late 1980s [49–51]. These initial examinations indicated that the Late Pleistocene strata at Kisese II include the southernmost examples of the dry grassland taxa Grevy’s zebra (*Equus grevyi*) and the extinct alcelaphine bovid *Damaliscus hypsodon* [51–53]. Impala (*Aepyceros melampus*) are present at Kisese II only in the uppermost 50 cm despite the fact that they are the dominant resident large mammal in nearby Tarangire National Park [37], implying key changes in regional habitats over time. Our current reconstructions of the Kisese II sequence began in 2011 and are based on Inskeep’s sparse published notes on the site [31, 42], material donated to us by his widow Adi, site photographs and artifact tallies retained by Inskeep now at the McDonald Archaeological Institute at Cambridge University, and new analyses of excavated lithic artifacts and fossil fauna stored at the National Museum of Tanzania (NMT) in Dar es Salaam, where they are un-numbered but accessible to other researchers.

Although our current focus is on the Pleistocene portions of the Kisese II sequence, the Holocene portions of the site contain several important aspects that we outline here. First, the painted slab that exfoliated from the wall and found by Inskeep was from the base of spit I. At least portions of spit I date to ~4 ka cal BP (discussed below), a reasonable maximum age estimate for the painted slab and visible rock art at the site by comparison with the sparse data from other shelters in the Kondoa region [30]. Spits I-II contain a broken *tuyère* and iron slag [42]. The site also preserves seven adult and infant burials, that, while undated, are likely Holocene in age, based on their preservation state and flexed position (cf. [17, 54]). Ceramic sherds from the Inskeep excavations stored at the NMT are undecorated and non-diagnostic but those from the uppermost portions of the adjacent Leakey excavations (also at the NMT) include < 2 ka Early Iron Age Lelesu Ware as well as < 5 ka Kansyore (LSA) sherds [55–58]. Spits I-II contain obsidian backed microliths (*n* = 25 at the NMT). Obsidian source(s) are undetermined. Mt. Kilimanjaro (~200 km) is the nearest known source, but geochemical analyses of obsidian artifacts from other northern Tanzanian LSA assemblages, including Kansyore strata at Mumba and Nasera rockshelters in northern Tanzania [17], indicate consistent use of sources near Lake Naivasha in central Kenya, ~400 km from Kisese II [59, 60]. Kisese II appears at or near the southernmost limit of the known distribution of Kansyore ceramics and non-local obsidian artifacts (cf. [55, 61]).

## Results

### Radiocarbon chronology

Although the stratigraphic position of the samples initially dated by the National Public Laboratory (UK) was not reported [44], Inskeep did provide the excavation level of the samples as a personal communication to Michael Mehlman, appearing in his 1989 Ph.D. thesis [17], which we report in Table 1. In order to better control the age and depositional history of the shelter, we generated 25 additional AMS ^14^C dates of the carbonate fraction of unburnt ostrich eggshell fragments of known stratigraphic position. Inskeep excavated the site in 28 ~15-cm-thick sub-horizontal spits or levels that were given sequential Roman numerals from top to bottom (Fig 1), and using collections curated at the NMT, we dated samples drawn from spits I-XXI (Table 1). No OES fragments were present below spit XXI [42], and the stratigraphically lowest clearly worked examples of OES beads occur in spit XX. We selected specimens for dating that showed no visible signs of human modification in order to maximize the sample of specimens for technological analyses of bead production. However, most of the dated pieces are likely initial stage (I/1) OES bead blanks [62–64] based on archaeological and ethnographic comparisons and the presence of partially worked specimens in all strata from spit XX and above. Regardless of their potential role in bead manufacture, all OES were likely transported to the site by humans and therefore their ages are indicative of human presence at Kisese II.

**Table 1:** Radiocarbon (^14^C) dates from Kisese II.

Spit	Laboratory code	δ^13^C	^14^C yr BP (±1σ)	calBP (95.4%)
I	UBA-27427*	-8.3	3,870 ± 30	4,410–4,160
	UBA-27428*	-9.0	3,840 ± 30	4,380–4,090
II	UBA-27430	-14.4	3,770 ± 20	4,230–3,990
	UBA-27429	-12.6	14,830 ± 60	18,190–17,840
III	UBA-27431	-12.6	14,270 ± 60	17,550–17,140
	UBA-27432	-10.7	14,020 ± 60	17,230–16,720
IV	NPL-35	–	14,760 ± 200	18,440–17,470
V	UBA-27433	-5.4	15,410 ± 70	18,810–18,510
	UBA-27434	-14.1	9,320 ± 40	10,650–10,300
	UBA-34477	-7.4	14,880 ± 60	18,250–17,900
VII	NPL-36	–	10,720 ±130	12,870–12,150
	UBA-34478	-8.4	38,040 ± 400	42,790–41,730
IX	NPL-37	–	18,190 ± 310	22,680–21,200
X	UBA-34479	-5.5	19,480 ± 80	23,680–23,130
XI	UBA-34480	-7.1	30,800 ± 220	35,140–34,250
XII	UBA-27435**	-15.2	30,620 ± 280	35,050–34,050
	UBA-27436	-9.7	30,070 ± 250	34,580–33,720
XIV	NPL-38	–	31,480 +1,640/-1,350	39,920–33,010
XV	UBA-27437	-10.1	30,930 ± 300	35,470–34,270
	UBA-27438**	-14.5	31,340 ± 290	35,850–34,660
XVIII	UBA-34481	-8.6	34,380 ± 290	39,590–38,340
XIX	UBA-34482	-6.9	27,790 ± 140	31,790–31,220
	UBA-34483	-10	36,740 ± 680	42,370–40,040
	UBA-27439	-10.7	33,420 ± 380	38,600–36,600
	UBA-27440	-7.3	41,200 ± 1,000	46,710–42,990
XX	UBA-34484	-9.6	40,600 ± 1,000	46,170–42,660
XXI	UBA-34485	-9.1	22,200 ± 120	26,740–26,070
	UBA-27441	-10.8	15,820 ± 60	19,230–18,890
	UBA-27442	-8.5	41,300 ± 1,000	46,850–43,120

All dates on the carbonate fraction of ostrich eggshell, calibrated using OxCal v.4.2 software [65] and a combined IntCal13/SHCal13 calibration curve [66, 67], reported at 95.4% probability.

* and ** indicate samples with overlapping δ^13^C values and calibrated radiocarbon dates, which could potentially derive from the same ostrich eggshell.

UBA are University of Belfast AMS dates on unburnt OES. NPL are National Public Laboratory conventional ^14^C dates on burnt OES [44]. δ^13^C values were measured for UBA samples by AMS to correct for instrument fractionation and have not been normalized to international standards (and therefore should not be used as stable isotopes values for inter-study comparisons). NPL-38 was calibrated using an estimated fraction of modern carbon (fM) of 0.0199+/-0.0037. Note that stratigraphic ordering within an excavation spit is undetermined.

We focused on OES fragments because charcoal, sediment, or other reliably dated materials are absent from the Inskeep collections. Bone is present throughout much of the stratigraphic sequence. The preservation of collagen suitable for radiocarbon dating from pre-Holocene deposits in the tropics is considered unlikely, although the development of inexpensive and minimally destructive screening techniques may change the feasibility of dating bone from Kisese II in the future [68]. However, the carbonate (inorganic) fraction of the shells of ostrich and other ratites has been shown to produce reliable dates using the AMS ^14^C method from tropical and sub-tropical settings in a number of studies [69–72]. In using OES to generate a sequence of dates throughout the Kisese II profile, we attempted to date several samples per spit to assess stratigraphic mixture, considered likely given Inskeep’s excavation in ~15-cm-thick horizontal spits and a paleotopography that variably dipped to the SE. However, the stratigraphic distribution of our dated samples is uneven (0–4 dated samples per spit) because among the OES curated at the NMT, not every spit is represented and some spits contained only burned or damaged specimens not considered suitable for dating. Furthermore, although each dated specimen can be attributed to an excavation spit, relative stratigraphic ordering of samples within the same ~15-cm-thick spit cannot be determined. We followed sample preparation methods of Janz and colleagues [69]. Dates >40 ka may reflect contaminant modern carbon and underestimate the true age of the specimens [73].

For calibration, OxCal 4.2 software [65] was used to model a mixed curve that combined the ranges of the northern (IntCal13) [66] and southern (SHCal13) [67] calibration curves. This approach increased the uncertainty of calibrated dates to account for the fact that Kisese II lies within the Inter-tropical Convergence Zone (ITCZ), where the hemispheric source of atmospheric CO_2_ has shifted seasonally and over time [74, 75]. Moreover, eggshell carbonate reflects local vegetation consumed by ostriches that roamed an unknown distance from the site [76–78]. Therefore it is unclear which curve to use, in what proportion, and calibrating by a combined curve provides a conservative range of possible dates. The code for our mixing model used in OxCal is provided in S1 Appendix.

**Fig 3:**
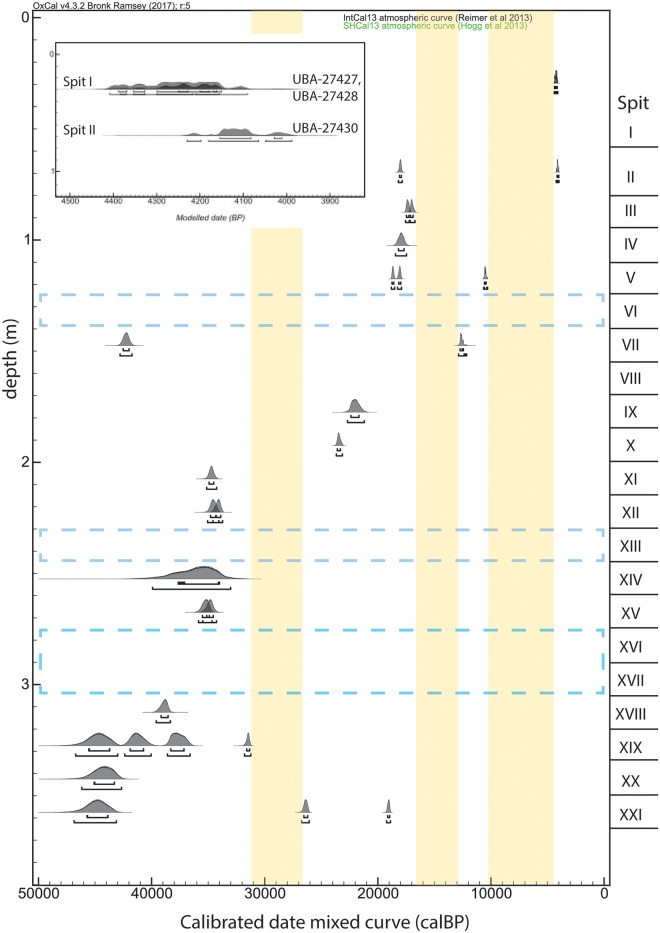
Age-depth relationship of ^14^C-dated samples. Calibrated radiocarbon dates are plotted by depth (estimated as midpoint of excavation spit). Blue dashed-line boxes indicate portions of the stratigraphic sequence with no dated specimens. Yellow bars indicate temporal spans with no measured radiocarbon dates. Dates calibrated using OxCal v.4.2 software [65] and a combined IntCal13/SHCal13 calibration curve [66, 67]. The insert shows the youngest dates from Spits I-II.

The new AMS and previously measured conventional ^14^C dates show good correspondence (Table 1). Sample ages generally increase with stratigraphic depth from ~4.0–46.9 ka cal BP (Fig 3). Only two pairs of specimens (UBA-27427/UBA-27428 and UBA-27435/UBA-27438) could potentially derive from the same eggshell, as the specimens in question have statistically indistinguishable AMS δ^13^C values and calibrated radiocarbon dates, determined by the Oxcal Combine Function [79]. We assume that several apparent outliers (UBA-34478, UBA-34485, UBA-27441) that differ in age from associated samples by over 15,000 years, do not represent the age of their stratigraphic position. The disagreements may be due to post-depositional mixing, contamination, or mistakes during excavation and storage. Disagreements between samples of less than 15,000 years (UBA-27434, UBA-34482) likely reflect the expected mixing of materials within a palimpsest excavated in horizontal levels that do not correspond to changes in sediment.

Bayesian models constrain the uncertainty of calibrated dates by incorporating known prior information, such as stratigraphic sequence, and quantitatively identify outliers [65, 79]. However, because the Kisese II samples come from collections of an old excavation, we cannot confirm their provenience beyond recorded excavation spits; that is, precise spatial coordinates are unavailable for the dated specimens. With this limited information, we produced several models in which spits were treated as phases (sequential, overlapping, with and without outlier analysis), but dates were not ordered within a given spit. The models failed to converge and had overall agreement indices between 0–5%. Models with agreement indices lower than 60% are general rejected [79]. A model with stronger priors was not justified.

There are several temporal gaps among the dated specimens (Fig 3), but it is unclear whether they should be attributed to lack of sampling in several spits, hiatuses in occupation, discontinuous sedimentation, or erosion. One such interval encompasses much of the early Holocene, roughly 10–4.5 ka cal BP. This period is regionally characterized by landscape stabilization and reduced sediment mobilization, as seen in the Lake Haubi basin ~30 km southeast of Kisese II [80], but local impacts at Kisese II remains to be determined. Despite limitations in sample distribution and clear instances of stratigraphic inversions within the sequence, the complete suite of new radiometric age estimates provides a reasonable chronology for the 47–4 ka cal BP interval preserved in the dated upper 3 m of the Kisese II sequence.

### Ostrich eggshell beads

The oldest clearly worked OES beads occur in spit XX, associated with one ^14^C sample dated to 46.2–42.7 ka cal BP. The first OES fragments of any kind are found in spit XXI, which contained at least one OES fragment dated to 46.9–43.1 ka cal BP. These dates confirm a general pattern of OES bead use at sites from the interior of eastern Africa [81], as they are comparable to but younger than the 52–47 ka range reported from Magubike and Mumba in Tanzania, and Enkapune ya Muto in Kenya. However, the significance of the Kisese II OES bead sample lies not in its age, but the size and temporal distribution of the sample. Inskeep recovered >5,000 complete OES beads, bead fragments and bead blanks discarded at various points in the production sequence. After their first clear appearance in spit XX, OES beads and associated bead production debris appears in every stratigraphic level through the sequence (Table 2), although material from some spits are now absent due to post-excavation loss.

**Table 2:** Summary data for Kisese II as reported by R.R. Inskeep (1956).

Spit	Backed pieces	Scrapers	Points	Misc. Modified Pieces	Cores	Lithic Total	OES beads	Spit volume (m^3^)
I	352	47	1	6	203	609	237	12.46
II	347	52	0	0	133	532	431	4.39
III	418	87	0	20	282	807	369	3.23
IV	314	78	1	17	286	696	543	3.23
V	134	59	3	28	204	428	673	3.23
VI	38	22	3	4	139	206	955	3.23
VII	29	33	0	3	105	170	822	3.23
VIII	85	62	1	25	149	322	354	3.23
IX	19	33	0	10	158	220	204	3.23
X	15	80	0	5	174	274	67	3.23
XI	5	50	0	4	73	132	195	3.23
XII	0	36	0	2	42	80	94	3.23
XIII	1	26	0	1	73	101	53	1.36
XIV	0	28	9	10	91	138	83	1.36
XV	1	39	12	19	96	167	89	1.36
XVI	1	19	0	38	55	113	31	1.36
XVII	4	34	0	55	70	163	40	1.36
XVIII	0	31	0	37	56	124	23	1.36
XIX	1	38	1	26	80	146	17	1.36
XX	1	27	3	18	94	143	9	1.36
XXI	2	11	0	8	56	77	0	1.36
XXII	1	24	0	5	66	96	0	1.36
XXIII	0	12	1	3	41	57	0	1.36
XXIV	2	7	0	0	44	53	0	1.36
XXV	1	6	0	1	18	26	0	0.64
XXVI	0	9	0	0	19	28	0	0.64
XXVII	0	5	0	0	9	14	0	0.64
XXVIII	0	8	0	2	25	35	0	0.64
Total	1771	963	35	347	2841	5957	5289	68.02

Summary artifact data for Kisese II from the 1956 excavation, based on tabulations from Inskeep's archives; counts for unmodified flakes, flake fragments, and debris are unknown. ‘OES beads’ includes OES bead fragments ranging from complete, finished beads to minimally modified fragments.

**Fig 4:**
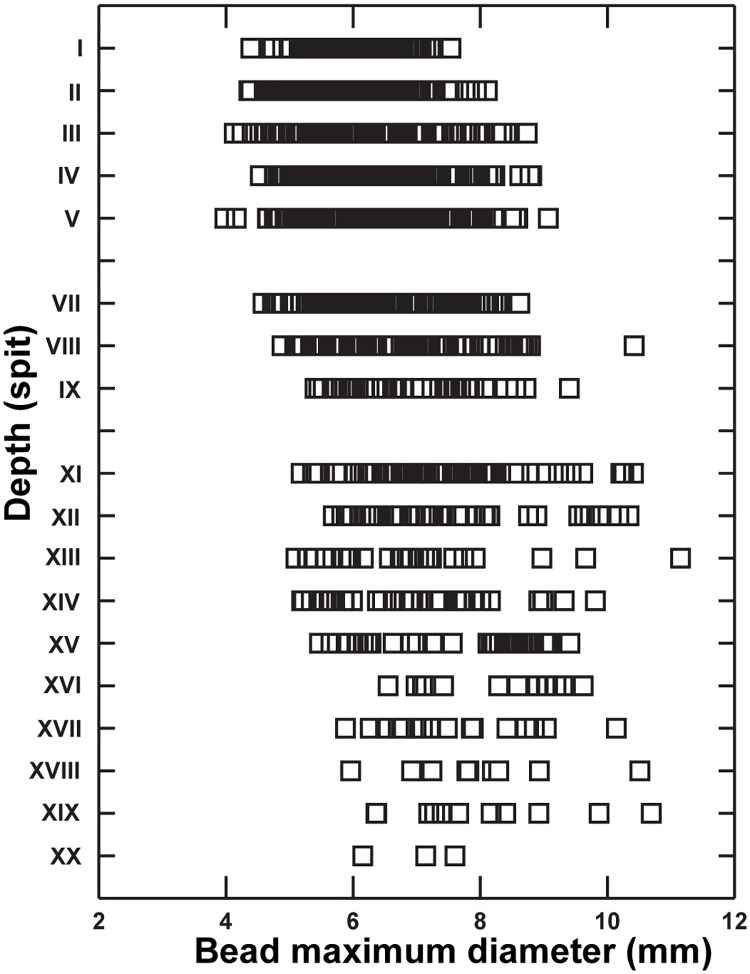
Temporal changes in ostrich eggshell bead size. Mean bead size (maximum diameter) declines over time at Kisese II in a sample of 1,400 specimens distributed over 20 excavated spits. Each square represents one bead.

The stratigraphic data indicate the repeated manufacture and use of OES beads at Kisese II. Metric data from the site suggest that this apparent uniformity in artifact type masks a continuous pattern of variation in bead morphology over time, coupled with variable patterns of production throughout the stratigraphic sequence. We measured all complete (phase 11/stage VII of [62, 63]) unburnt ostrich eggshell beads (*n* = 1,400) housed at the NMT, summarized in Table 3. Bead maximum diameter varies across the stratigraphic sequence (F (2,17) = 24.675, p < 0.001), with mean bead size increasing with depth (*r*_*s*_ = 0.895, *p* < 0.001), from ~6.5 mm near the top of the sequence to ~7.5 mm near the base (Fig 4). The scale of change is small (≤ 1 mm), spread across > 40 kyr, and as bead size co-varies with aperture size (*r* = 0.654, *p* < 0.001), changes in size might simply reflect shifts in drilling technology rather than deliberate selection for smaller beads over time (see [82]). In East Africa, finely tipped stone drills (or ‘microperçoirs’) appear to be present only among LSA deposits (e.g., [17, 83]). The pattern of *decreased* bead size over time at Kisese II is supported by a comparison with 10 other East African sites for which metric data are available (Table 3). This extended sample lacks the size or chronological resolution of the Kisese II data, but also indicates the presence of significantly smaller beads in the Holocene than in the Pleistocene (*z* = -10.051, *p* < 0.001). The large sample (*n* = 1,780) of complete OES beads from Bed III at Mumba [84] is not included in this comparison because an unconformity spans the Pleistocene-Holocene boundary there, which, combined with other taphonomic issues with the Bed III deposits [17, 85] make chronological attribution of the analyzed sample difficult. The lack of stratigraphic differences in bead size within the Mumba Bed III sample (all are ~6.7 mm in diameter) [84] emphasizes the importance of long sequences like Kisese II for recognizing subtle temporal trends.

**Table 3:** Descriptive statistics of ostrich eggshell beads from Kisese II and other East African archaeological sites.

Site	Stratigraphic level	Age estimate (ka)	*n*	Diameter (mm)	Diameter range (mm)	Diameter CV (%)	Aperture size (mm)	Aperture CV (%)
Kisese II	I	4.2	104	6.0±0.6	4.4–7.5	10.0	2.4±0.4	16.7
Kisese II	II	18.1–4.2	162	6.0±0.8	4.4–8.1	13.3	2.2±0.4	18.2
Kisese II	III	17.5–16.7	135	6.2±1.0	4.1–8.7	16.1	2.4±0.4	16.7
Kisese II	IV	18.4–17.4	205	6.3±0.9	4.5–8.8	14.3	2.4±0.5	20.8
Kisese II	V	18.8–17.9	195	6.4±1.0	4.0–9.1	15.6	2.4±0.5	20.8
Kisese II	VI	22.7–17.9	N.D.	N.D.	N.D.	N.D.	N.D.	N.D.
Kisese II	VII	22.7–17.9	179	6.5±0.9	4.6–8.6	13.8	2.7±0.5	18.5
Kisese II	VIII	22.7–17.9	84	6.8±1.1	4.9–10.4	16.2	2.7±0.5	18.5
Kisese II	IX	22.7–21.2	44	6.8±1.0	5.4–9.4	14.7	3.0±0.4	13.3
Kisese II	X	23.7–23.1	N.D.	N.D.	N.D.	N.D.	N.D.	N.D.
Kisese II	XI	35.1–34.3	74	7.4±1.1	5.2–10.4	14.9	2.8±0.7	25.0
Kisese II	XII	35.1–33.7	41	7.4±1.3	5.7–10.3	17.6	2.9±0.6	20.7
Kisese II	XIII	39.9–33.7	28	6.9±1.4	5.1–11.2	20.3	2.4±0.4	16.7
Kisese II	XIV	39.9–33.0	41	6.8±1.3	5.2–9.8	19.1	2.7±0.6	22.2
Kisese II	XV	35.9–34.3	51	7.9±1.1	5.5–9.4	13.9	3.1±0.5	16.1
Kisese II	XVI	39.6–34.3	14	8.3±1.0	6.6–9.6	12.0	3.2±0.5	15.6
Kisese II	XVII	39.6–34.3	20	7.6±1.1	5.9–10.1	14.5	3.1±0.6	19.4
Kisese II	XVIII	39.6–38.3	9	8±1.3	6.0–10.5	16.3	3.2±0.7	21.9
Kisese II	XIX	46.7–31.2	11	8±1.4	6.4–10.7	17.5	2.9±0.8	27.6
Kisese II	XX	46.2–42.7	3	7±0.7	6.2–7.6	10	2.7±0.4	14.8
GvJm16 [86, 87]	L.S. Bed C	Holocene	7	6.3±0.3	5.9–6.7	4.8	2.6±0.3	11.5
GvJm16 [86, 87]	U.S. Bed B	Holocene	15	6.8±0.4	5.9–7.4	5.9	2.8±0.4	14.3
GvJm22 [15, 88]	Occurrence D	6.7–3.7	5	6.4±0.4	5.8–6.7	6.3	2.4±0.6	25.0
GvJm22 [15, 88]	Occurrence E	22–15	1	7.52	N.A.	N.A.	3.2	N.A.
Gamble's Cave II [89, 90]	4^th^ occ. level	10–9	63	5.6±0.8	4.0–8.2	14.3	1.9±0.4	21.1
Porcupine Cave [91]	Layers 1–3	3–0	7	6.9±0.9	6.2–8.2	13.0	2.1±0.2	9.5
Porcupine Cave [91]	Layer 5	> 3	16	6.3±0.6	5.6–7.8	9.5	2.1±0.4	19.0
Enkapune ya Muto [81, 92]	DBL	46–42	7	7.6±1.0	6.7–9.5	13.2	2.9±0.6	20.7
Mlambalasi [19, 93]	Levels 1–10	12–0	50	6.4±1.3	2.5–8.9	20.3	2.3±0.6	26.1
Magubike [81]	40–50 cm	16–15	1	6.6	N.A.	N.A.	3.1	N.A.

All measurements of OES beads are on complete, finished, unburnt beads (phase 11/stage VII of [62, 63]), and listed as mean and one standard deviation. Published metric data were used for Mlambalasi, with sizes for beads from Magubike and Enkapune ya Muto estimated from published photographs and illustrations; all other metric data collected by the authors from specimens housed at the Tanzanian National Museum or the National Museums of Kenya. Absence of beads from spits VI and X at Kisese II likely reflect post-excavation sample loss. L.S., Lower Shelter; U.S., Upper Shelter; N.D., no data; N.A., not applicable. CV, coefficient of variation (sample standard deviation divided by sample mean), expressed as a percentage.

**Fig 5:**
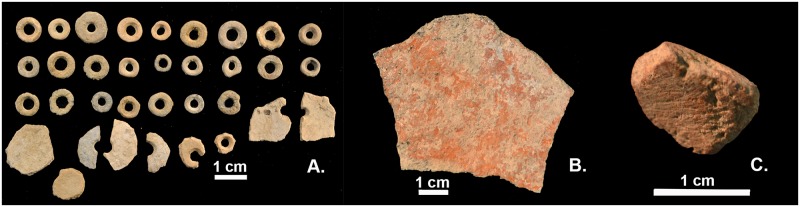
Ostrich eggshell beads and ochre. A. Ostrich eggshell beads, bead blanks, and production debris from Kisese II spit XVII at the National Museums of Tanzania, showing diversity of production methods, B. ochre ‘palette’ from spit III and C., ochre piece with striations from use (spit VI). Photographs by C. Tryon and K. Ranhorn.

Despite general similarities in the form of the finished bead, the methods of OES bead production appear variable at Kisese II. Technological analyses of bead production methods have not been conducted on the complete Kisese II sample, but the sample includes beads that were manufactured by a variety of approaches documented ethnographically and experimentally [62–64, 87, 93–95], even among samples drawn from a single excavated spit (Fig 5). These include the drilling of angular fragments, the creation of rounded disks prior to perforation, and drilling done from the exterior, interior, or both sides of the shell. However, restricted metric variation within Holocene samples from Kisese II and other East African sites suggest temporal shifts in social expectations or production practices. The coefficient of variation (CV) of bead diameter (Table 3) for Holocene samples is significantly lower than Late Pleistocene ones (*z* = -2.535, *p* = 0.011), overlapping with values seen among craft specialists in other contexts [96, 97]. The extent to which this more restricted bead size relates to a narrower range of production methods remains unresolved in East Africa (cf. [63]).

### Ochre and grinding stones

Ochre, useful as a pigment to color bodies, walls, and beads, as a binder, and for a number of other purposes [98–101], was being transported to and processed at Kisese II before ~45 ka cal BP, and appears to be a common feature among the painted shelters of the Kondoa Rock-Art UNESCO World Heritage Center [30–33, 102]. Inskeep (in [31]) reports red (and rare orange and yellow) ochre in all Kisese II strata from spit XXVI (>43 ka cal BP) upwards, and red ochre was used to make many of the extant paintings at the site. The 1–203 g per spit from the Inskeep collections at the NMT today are minimum abundance estimates of past usage, based on the larger quantities of ochre from the adjacent Leakey excavations, which have a different curation history and may more accurately reflect the amount of material recovered. Ochre pieces with wear-facets are present from spit XXIII (>43 ka cal BP) and up. Stone tools showing evidence for grinding or smoothing from use, including stone ‘palettes’ with ochre-stained surfaces (Fig 5), first appear in spit XVII [31], which is bracketed by spits dated from 39.6 ka cal BP to 34.3 ka cal BP. Ochre-stained stone ‘palettes’ appear intermittently in overlying strata, although their precise temporal distribution at the site cannot now be reconstructed with certainty.

The sources of the ochre used at Kisese II and other nearby painted shelters are unknown. Local sources likely exist and may have been used, given the abundance of ochre at archaeological and painted sites in Kondoa. One probable source are the iron-rich sediments that provided the raw material for iron smelting at Kisese II and other Iron Age and more recent sites in the region [42, 57, 58, 103].

### Middle and Later Stone Age lithic technology

**Fig 6:**
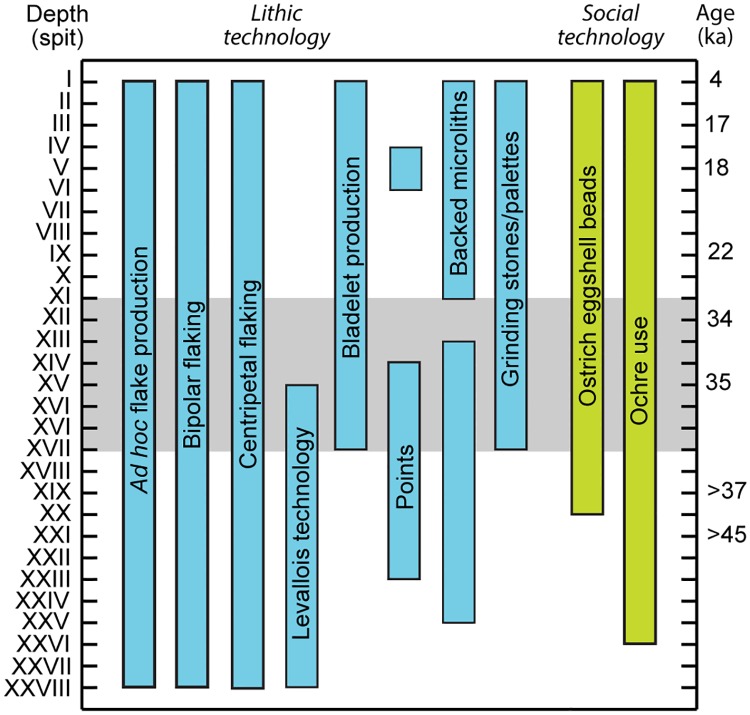
Synthesis of temporal trends of archaeological change at Kisese II. First and last appearance datums for particular artifact types or technologies are reconstructed from archival and published data as well as new analyses of material stored at the National Museums of Tanzania in Dar es Salaam. Grey shading arbitrarily denotes an interval with multiple changes that may record the Middle/Later Stone Age transition.

The Kisese II lithic assemblage shows a number of qualitative and quantitative changes that suggests a sequence that spans the MSA/LSA transition. The presence of MSA and LSA artifacts is consistent with our new chronology for Kisese II compared to other East African sites considered to sample both MSA and LSA deposits (reviewed in [3]). The most relevant comparisons are sites with relatively long stratigraphic sequences from similar environmental settings, particularly Nasera [8, 17, 104, 105] and Mumba [17, 18, 21, 85, 105–107] in northern Tanzania and multiple sites at Lukenya Hill in southern Kenya [15, 86, 108, 109], but also Enkapune ya Muto (Kenya) [92], Mlambalasi and Magubike (Tanzania) [19, 110], and perhaps Magosi (Uganda) [16, 111, 112] (Fig 1). We restrict our discussion of the Kisese II lithic material to general statements about temporal trends, summarized in Fig 6, and emphasize the presence, more than the absence, of particular raw materials, types, or technologies. We do this because of the nature of the available sample. Inskeep’s archival notes indicate that he analyzed 5,048 retouched tools and cores, listed in Table 2, but the number of unmodified flakes, flake fragments and debris was apparently unrecorded. The sample that remains at the NMT in Dar es Salaam is considerably smaller at ~2,252 total pieces, but includes both retouched tools and cores *and* unmodified pieces. This indicates that a minimum of 56% of the assemblage is currently unavailable for study.

As noted by Inskeep and visible among the NMT collections, the Kisese II lithic assemblage consists of flakes, cores, and retouched pieces made almost entirely on locally available coarse-grained to crystalline quartz from veins and stream pebbles. Chert and lava artifacts are rare. Chert sources are unknown; the nearest source of lava is the Pleistocene nephelinite/carbonatite volcano Kwaraha ~30 km to the north [113]. A single obsidian bipolar core comes from spit XIV. As with other quartz-based lithic assemblages in sub-Saharan Africa (e.g., [17]), there is evidence for freehand direct percussion using an *ad hoc* approach that resulted in single- and multi-platform cores throughout the stratigraphic sequence. Pieces indicative of bipolar percussion, including bipolar cores and *outils écaillés*, are also found throughout the stratigraphic sequence.

**Fig 7:**
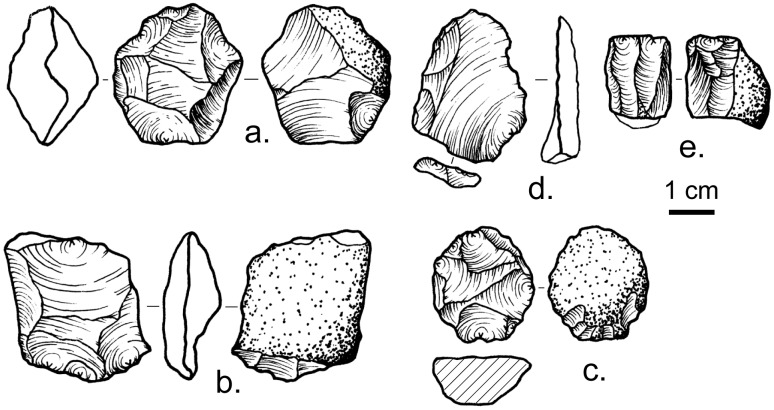
Lithic artifacts from Kisese II. a., discoidal core from spit XVIII, b.-c., recurrent Levallois cores from spits XIX and XX, respectively, d., retouched flake, possible Kombewa flake from split-pebble core from spit XVI, e., single platform bladelet core from spit XV. All artifacts are quartz, except c., which is chert. Illustrations are 1:1 scale.

Other flake production technologies appear to show more restricted stratigraphic distributions. In lower strata (spits XXVIII-XVIII) likely dating from >43 ka cal BP to 38.3 ka cal BP, scar patterns on cores and flakes document use of a broad spectrum of reduction methods that share centripetal or ‘radial’ patterns of flake removal. Cores from these centripetal reduction methods in the lower strata include discoidal (Fig 7a) and recurrent Levallois forms (Fig 7b and 7c). ‘Semi-radial pebble cores’ [114], split pebbles with multiple centripetal removals on the initial fracture surface, also occur in these levels, with some Kombewa flakes (cf. [115, 116, 117]) made in the course of their reduction (Fig 7d). Levallois and semi-radial pebble cores both preserve a hierarchy to the flake removal surfaces that is characteristic of prepared core methods [118], and represent a typical MSA flake production technology [3]. Spits XVII-XV (39–34.3 ka cal BP) show a continuation of the pattern of centripetal reduction seen in the lower strata, but also contain small (< 2 cm) single and opposed platform cores for bladelet production (Fig 7e), a typical LSA technology in East Africa [114]. This suggests the co-occurrence of the characteristic MSA and LSA technologies. Based on the sample at the NMT, by spit XIII (bound by spits dated from 35.8–33.7 ka cal BP), Levallois technology is absent, although discoidal methods persist, and by spit VIII dorsal scars on many of the platform and bipolar cores indicate bladelet production. Spit VIII is imprecisely dated, but likely in the 22–18 ka cal BP range.

In terms of retouched tools, we emphasize the presence of retouched points and backed microliths because they are considered characteristic implements of the East African MSA and LSA respectively. The Inskeep archives (Table 2) indicate that retouched points first appear in spit XXIII, and are most common in spits XV-XIV (*n* = 21) dated to ~35.8–33.0 ka calBP, with a second, smaller concentration in spits VI-V (*n* = 6), which we estimate to date to 22–18 ka cal BP. No retouched points are present among the material from Inskeep’s excavations in Dar es Salaam and we cannot confirm his typological attributions. Backed pieces are present in small numbers (*n* = 1–2) in some levels from spits XXV-XIII (>43 ka cal BP to ~39.9–33.7 ka cal BP), and starting with spit XI (35.1–34.3 ka cal BP) are present in every level in increasingly larger quantities (Table 2). In spit VIII and above, backed microliths are the most abundant type of retouched tool (Table 2), outnumbering scrapers, which are otherwise the most common formal tool type at Kisese II, occurring throughout the stratigraphic sequence (Table 2). The pattern of incremental increases in backed piece frequency is also seen at Mumba and Nasera [17], with backed pieces as the dominant type of retouched tool at other Last Glacial Maximum (LGM) and post-LGM Late Pleistocene LSA assemblages in the East African interior, particularly at Lukenya Hill [108, 109] and also at Munyama Cave (Uganda) [119].

## Discussion

Fig 6 documents the results of our archival- and collections-based reconstructions of stratigraphic patterning in the presence, absence, and persistence of particular types of artifacts and ways of making them at Kisese II. These data, combined with our estimate of the age of the site’s sequence, suggest that Kisese II preserves MSA and LSA assemblages, the latter including pottery and traces of iron working in the uppermost late Holocene (~4 ka) deposits. Evidence for some behaviors are present throughout much of the site’s history, with first appearances for them well beyond the limit of the radiocarbon method, including *ad hoc*, bipolar, and centripetal approaches to core reduction, as well as the use of ochre. Ostrich eggshell beads first appear in spit XX dated to 46.2–42.7 ka cal BP, with possible bead blanks in spit XXI dated as early as 46.9–43.1 ka cal BP. A number of changes appear to occur between spits XVII-XI, including the disappearance of Levallois technology and (at least temporarily) the abandonment of point manufacture, as well as the first evidence for bladelet production and the use of grinding stones or palettes. Backed microliths appear early in the sequence in spit XXV, estimated at >43 ka cal BP and occur infrequently in overlying strata, including between spits XVII-XI, becoming abundant only in spit X, dated to ~23.7–23.1 ka cal BP. Because the directly dated OES fragments (Table 1) indicate the downward migration of some small pieces through the sediment column, the true first appearance date of backed microliths at Kisese II is poorly defined. If spits XVII-XI at Kisese II can be interpreted as the MSA/LSA ‘transition’ on the basis of lithic technology, then dates from spits XVIII and XI indicate a minimum age range of 39.6–34.3 ka cal BP for this process. The actual duration of the shift to fully LSA technologies at Kisese II may be considerably longer, perhaps beginning beyond the ^14^C limit based on the presence of rare backed pieces, and possibly continuing into the Last Glacial Maximum, given the ~23 ka cal BP dated sample from overlying spit X.

There is a well-documented emphasis on origins or first appearance datums (FADs) in the historical sciences in general and paleoanthropology in particular (e.g., [120]). In East Africa, early appearance dates (~55–45 ka) for backed microliths and ostrich eggshell beads at sites such as Enkapune ya Muto [92] and Mumba [20] have received considerable attention because of their perceived importance as markers of modern human behavior and their broad chronological overlap with the timing of the Middle/Upper Paleolithic transition in western Eurasia and human dispersals out of Africa (e.g., [2, 121, 122]). While useful, an emphasis on FADs tends to downplay evolution *within* technical systems, and obscure the processes by which innovations are spread from their point of origin; they also mask the potential to examine the loss and reinvention of technology and to detect convergent evolution among disparate groups.

The MSA/LSA transitional assemblages from Kisese II, which date to at least 39–34.3 ka cal BP, are younger than the MSA Endingi, and LSA Nasampolai and Sakutiek industries at Enkapune ya Muto [92] and the Mumba Industry at Mumba, considered by some to be an early LSA entity [18, 107]. However, dates similar to those from Kisese II MSA/LSA transitional assemblages have been reported for the MSA/LSA Nasera Industry at Mumba, dated by optically stimulated luminescence to 36.8±3.4 ka [20]. MSA artifacts from the Lake Victoria basin in Kenya overlie and are therefore younger than a volcanic ash dated by ^40^Ar/^39^Ar to 35.62±0.26 ka [123, 124]. The Kisese II data appear to indicate that the shift to LSA technologies was a time-transgressive process in East Africa; that is, the first appearance datums of the LSA and the elements that define it differ among sites in the region. Therefore, the FADs from Enkapune ya Muto and Mumba cannot be considered ‘isochrons’ applicable to all sites across East Africa. More extreme examples of the ‘late’ persistence of technologies are seen in the Horn of Africa, where sites such as Goda Buticha [125, 126] and K’aaba and Bel K’urk’umu [127] appear to preserve typical MSA elements such as points and Levallois technology well into the Holocene. The data from East Africa and the Horn appear to reflect a larger pattern in sub-Saharan Africa for the variable and often late persistence of MSA technologies considerably younger than 45 ka, as reported for western Africa [128], southeastern Africa [129, 130] and the southern African interior [131]. Determining whether the temporal variation in these MSA/LSA shifts is due to independent trajectories of technological evolution related to changes in local habitats or population density, or the delayed diffusion of ideas or populations over time remains a key focus for future research.

The Kisese II chronological and archaeological data provide a basic framework to understand the timing, tempo, and nature of behavioral changes across the MSA/LSA transition. Changes in social technologies (OES beads) at least ~43 ka cal BP precede shifts in subsistence technologies (flaked stone artifacts) that span at least 39–34.3 ka cal BP. These are robust data for understanding the timing of the MSA/LSA transition, and suggest a tempo for the ‘transition’ at minimum 5–10 kyr. The nature of the changes appears to be incremental, with new technologies appearing across more than a meter of sediment, but this represents the limits of the resolution afforded by available archival and museum collections. The extent to which the patterns seen at Kisese II are a result of thick, arbitrary units of excavation [85, 107] or later bioturbation can only be addressed through additional fieldwork, microstratigraphic approaches, and careful assessment of post-depositional processes by artifact refitting [132, 133] and allied approaches.

## Conclusions

The Kisese II rockshelter is a locally prominent landscape feature used recurrently throughout portions of the Late Pleistocene and Holocene. Initial excavation at the site in the 1950s by L.S.B. and M.D. Leakey and later R. Inskeep indicated the archaeological importance of the rockshelter. At a minimum, Kisese II functioned as a locus for the manufacture, maintenance, or use of stone tools, an area where ochre and beads were worked, and by the Holocene if not before, as a place to bury the dead and as a surface to paint. Despite the site’s importance, limited publication of the excavation results has caused it to play at best a minor role in discussions of the archaeology of East Africa or the behavior of Pleistocene *Homo sapiens* more generally. Using archival materials and artifact collections in the UK and Tanzania, our research efforts have focused on reversing this situation, by providing an examination and re-contextualization of the 1950s excavations at Kisese II as a necessary prelude to our renewed excavations at the site.

As a result of this work, the upper 3-m of the >6-m-thick archaeological sequence at Kisese II rockshelter is dated to 47–4 ka cal BP by a suite of 25 new AMS ^14^C determinations on the carbonate fraction of ostrich eggshell (OES) fragments. OES fragments were targeted because they were available, are appropriate material to date from museum collections, and were almost certainly introduced to the shelter by humans. Additional chronological control on stratigraphically lower parts of the site will require other techniques such as optically stimulated luminescence or electron spin resonance that need *in situ* sediment for dating or dosimetry, accessible only with further excavations at the site. The renewed analysis of the artifacts indicates the recurrent use of ochre (first appearing >43 ka cal BP) and abundant ostrich eggshell beads (first appearing 46.2–42.7 ka cal BP) throughout the site’s history, the latter diminishing in size over time.

The Holocene deposits contain a variety of LSA and Iron Age ceramic types. Pleistocene strata include MSA artifact forms such as pieces showing Levallois methods of flake production and possible retouched points, as well as typical LSA implements such as backed microliths and bladelets. This suggests that the site spans the MSA/LSA transition, with a number of important lithic technological changes occurring 39–34.3 ka cal BP, and extending perhaps to the Last Glacial Maximum. While the MSA/LSA transition is often implicated in discussions of modern human origins, our results support the hypothesis that the timing of this process varied even within a single region in Africa. Determining the extent to which this variation is a result of human population dispersals within Africa, the diffusion of new technologies among social networks, or local adaptations to changing environments or population densities requires considerably more work at Kisese II and sites like it.

## Supporting information

S1 Appendix Code used in OxCal 4.2 [65] software to produce a mixed model for radiocarbon dates incorporating both the northern hemisphere (IntCal13) and southern hemisphere (SHCal13) calibration curves [66, 67]. (DOCX)
