# Supplementary material for: Middle and Later Stone Age chronology of Kisese II rockshelter (UNESCO World Heritage Kondoa Rock-Art Sites), Tanzania
Source: PLoS One. 2018 Feb 28;13(2):e0192029. doi: 10.1371/journal.pone.0192029 (PMC5830042; doi:10.1371/journal.pone.0192029)
Supplement: S1 Appendix — (DOCX) [file pone.0192029.s001.docx]

**S1 Appendix**

The following code was used in OxCal 4.2 [1] software to produce a mixed model incorporating both the northern hemisphere (IntCal13) and southern hemisphere (SHCal13) calibration curves [2, 3].

Plot()
 {
  Curve("IntCal13","IntCal13.14c");
  Curve("SHCal13","SHCal13.14c");
  Mix_Curve("Mixed","IntCal13","SHCal13",U(0,100));
  Curve("=Mixed");
  R_Date("UBA-27427", 3873, 27)
  {
   z=0.295;
  };
  R_Date("UBA-27428", 3838, 27)
  {
   z=0.295;
  };
  R_Date("UBA-27430", 3772, 24)
  {
   z=0.695;
  };
  R_Date("UBA-27429", 14825, 57)
  {
   z=0.695;
  };
  R_Date("UBA-27431", 14273, 62)
  {
   z=0.875;
  };
  R_Date("UBA-27432", 14016, 62)
  {
   z=0.875;
  };
  R_Date("NPL-35", 14760, 202)
  {
   z=1.025;
  };
  R_Date("UBA-27433", 15410, 67)
  {
   z=1.175;
  };
  R_Date("UBA-27434", 9322, 38)
  {
   z=1.175;
  };
  R_Date("UBA-34477", 14882, 55)
  {
   z=1.175;
  };
  R_Date("NPL-36", 10720, 132)
  {
   z=1.475;
  };
  R_Date("UBA-34478", 38037, 396)
  {
   z=1.475;
  };
  R_Date("NPL-37", 18190, 306)
  {
   z=1.775;
  };
  R_Date("UBA-34479", 19476, 79)
  {
   z=1.925;
  };
  R_Date("UBA-34480", 30800, 223)
  {
   z=2.075;
  };
  R_Date("UBA-27435", 30618, 277)
  {
   z=2.225;
  };
  R_Date("UBA-27436", 30065, 254)
  {
   z=2.225;
  };
  R_F14C("NPL-38", 0.0199, 0.0037)
  {
   z=2.525;
  };
  R_Date("UBA-27437", 30932, 296)
  {
   z=2.675;
  };
  R_Date("UBA-27438", 31335, 292)
  {
   z=2.675;
  };
  R_Date("UBA-34481", 34381, 293)
  {
   z=3.125;
  };
  R_Date("UBA-34482", 27786, 138)
  {
   z=3.275;
  };
  R_Date("UBA-34483", 36735, 680)
  {
   z=3.275;
  };
  R_Date("UBA-27439", 33423, 377)
  {
   z=3.275;
  };
  R_Date("UBA-27440", 41157, 1019)
  {
   z=3.275;
  };
  R_Date("UBA-34484", 40608, 1027)
  {
   z=3.425;
  };
  R_Date("UBA-34485", 22198, 119)
  {
   z=3.575;
  };
  R_Date("UBA-27441", 15819, 64)
  {
   z=3.575;
  };
  R_Date("UBA-27442", 41310, 997)
  {
   z=3.575;
  };
 };

1. Bronk Ramsey C. Bayesian analysis of radiocarbon dates. Radiocarbon. 2009;51:337-60.

2. Reimer PJ, Bard E, Bayliss A, Beck JW, Blackwell PG, Bronk Ramsey C, et al. IntCal13 and Marine13 radiocarbon age calibration curves 0-50,000 years cal BP. Radiocarbon. 2013;55:1869-87.

3. Hogg AG, Hua Q, Blackwell PG, Niu M, Buck CE, Guilderson TP, et al. SHCal13 Southern Hemisphere Calibration, 0–50,000 Years cal BP. Radiocarbon. 2013;55(4):1889-903. Epub 02/09. doi: 10.2458/azu_js_rc.55.16783.
